# Additional Insights Into the Analgesia Management of a High-risk Postoperative Setting

**DOI:** 10.4274/TJAR.2026.262758

**Published:** 2026-10-30

**Authors:** Rohan Magoon, Nitin Choudhary

**Affiliations:** 1 Atal Bihari Vajpayee Institute of Medical Sciences (ABVIMS) and Dr. Ram Manohar Lohia Hospital, Department of Anaesthesia and Cardiac Anaesthesia, Baba Kharak Singh Marg, New Delhi, India; 2 All India Institute of Medical Sciences (AIIMS), Department of Anaesthesiology, Pain Medicine and Critical Care, Sri Aurobindo Marg, New Delhi, India

**Keywords:** Cardiovascular and thoracic anaesthesia, intensive care, pain, perioperative care, pharmacology, regional anaesthesia

To the Editor,

Akın et al.^1^ are sincerely commended on formulating an effective analgesia management plan centered around the use of interfascial plane blocks in their high-risk patient who had undergone concomitant liver transplant and coronary artery bypass grafting (CABG) surgery in a single surgical setting. Meanwhile, the combination of pecto-intercostal fascial plane block (PIFB) and modified thoracoabdominal nerve block via perichondrial approach aided the postoperative stabilization and successful extubation of the index patient; however, other important factors warrant elaboration.^1^

To begin with, Akın et al.^1^ employed a total of 80 mL of 0.125% bupivacaine for administering the interfascial blocks where no rescue analgesia was interestingly required amidst satisfactory pain scores for the entire duration of 24 h, despite the absence of an added adjuvant to the local anaesthetic (LA) mixture. While duly acknowledging the authors’ caution not to exceed the maximum safe dose for the corresponding LA, we note that the choice of LA agent deserves attention.^1, 2^ Notably, the literature suggests that ropivacaine is less cardiotoxic than bupivacaine, even at equipotent doses.^2^ The former becomes more important in the context of a CABG patient when pre-operative and post-operative left ventricular ejection fraction data are missing.^1^ The predisposition to postoperative rhythm disturbances should not be overlooked, particularly in high-risk postoperative settings.^1, 2^ Secondly, a Delphi consensus led by “American Society of Regional Anesthesia and Pain Medicine” and the “European Society of Regional Anaesthesia and Pain Therapy” recommends employing anatomically descriptive nomenclature in regional analgesia.^3, 4, 5^ It is recommended that PIFB be referred to as the superficial parasternal intercostal plane block for standardized academic and clinical communication.^3, 4^ Finally, given the pivotal role of multimodal analgesia in assisting enhanced recovery after surgery, the formal account of the quality of recovery scores alongside the 24 h pain scores would have added incremental objective value to the index case presentation.^1, 5^
